# Nasopharyngeal Cancer Incidence and Mortality in 185 Countries in 2020 and the Projected Burden in 2040: Population-Based Global Epidemiological Profiling

**DOI:** 10.2196/49968

**Published:** 2023-09-20

**Authors:** Yanting Zhang, Harriet Rumgay, Mengmeng Li, Sumei Cao, Wanqing Chen

**Affiliations:** 1 Department of Epidemiology and Health Statistics, School of Public Health, Guangdong Medical University Dongguan China; 2 Cancer Surveillance Branch, International Agency for Research on Cancer Lyon France; 3 Department of Cancer Prevention, Sun Yat-sen University Cancer Center Guangzhou China; 4 State Key Laboratory of Oncology in South China, Guangdong Key Laboratory of Nasopharyngeal Carcinoma Diagnosis and Therapy, Sun Yat-sen University Cancer Center Guangzhou China; 5 Office of Cancer Screening, National Cancer Center of China/Cancer Hospital, Chinese Academy of Medical Sciences and Peking Union Medical College Beijing China

**Keywords:** nasopharyngeal cancer, incidence, mortality, epidemiology, worldwide

## Abstract

**Background:**

Nasopharyngeal cancer (NPC) is one of the most common head and neck cancers.

**Objective:**

This study describes the global epidemiological profiles of NPC incidence and mortality in 185 countries in 2020 and the projected burden in 2040.

**Methods:**

The estimated numbers of NPC cases and deaths were retrieved from the GLOBOCAN 2020 data set. Age-standardized incidence rates (ASIRs) and age-standardized mortality rates (ASMRs) were calculated using the world standard. The future number of NPC cases and deaths by 2040 were estimated based on global demographic projections.

**Results:**

Globally, approximately 133,354 cases and 80,008 deaths from NPC were estimated in 2020 corresponding to ASIRs and ASMRs of 1.5 and 0.9 per 100,000 person-years, respectively. The largest numbers of both global cases and deaths from NPC occurred in Eastern Asia (65,866/133,354, 49.39% and 36,453/80,008, 45.56%, respectively), in which China contributed most to this burden (62,444/133,354, 46.82% and 34,810/80,008, 43.50%, respectively). The ASIRs and ASMRs in men were approximately 3-fold higher than those in women. Incidence rates varied across world regions, with the highest ASIRs for both men and women detected in South-Eastern Asia (7.7 and 2.5 per 100,000 person-years, respectively) and Eastern Asia (3.9 and 1.5 per 100,000 person-years, respectively). The highest ASMRs for both men and women were found in South-Eastern Asia (5.4 and 1.5 per 100,000 person-years, respectively). By 2040, the annual number of cases and deaths will increase to 179,476 (46,122/133,354, a 34.58% increase from the year 2020) and 113,851 (33,843/80,008, a 42.29% increase), respectively.

**Conclusions:**

Disparities in NPC incidence and mortality persist worldwide. Our study highlights the urgent need to develop and accelerate NPC control initiatives to tackle the NPC burden in certain regions and countries (eg, South-Eastern Asia, China).

## Introduction

### Background

Nasopharyngeal cancer (NPC) is an epithelial carcinoma that arises from the mucosal lining of the nasopharynx. NPC is one of the most common head and neck cancers and is characterized by remarkable geographic variation. Historically, NPC incidence rates were less than 1 per 100,000 person-years in most parts of the world, but they have been higher than 20 per 100,000 person-years in South-Eastern Asia and Southern China in the past decades [1-3]. In contrast, NPC incidence and mortality rates in most countries worldwide have significantly decreased over the past decades [4,5]. Notably, NPC is usually diagnosed in advanced stages and has a very poor prognosis [6]. For example, the 5-year net survival is estimated to be 47% in China [7].

### Risk Factors Associated With NPC Occurrence

NPC is a largely preventable disease due to its many modifiable risk factors. The major etiological factors for NPC include Epstein-Barr virus (EBV) infection, tobacco smoking, intake of salted fish and other salt-preserved food, and occupational exposure to wood dust [8]. EBV infection has been consistently identified as an important risk factor, with a dose-response relationship between EBV antibody level and NPC risk [9,10]. The population attributable fraction for NPC incidence due to EBV infection has been estimated to be 85% [11]. Compared with people who never smoke, people who formerly smoked have a 60% greater risk of developing NPC [12]. The relative risk of NPC associated with weekly consumption of Chinese-style salt-preserved fish ranges from 1.1 to 4, whereas that associated with daily consumption ranges from 1.8 to 20 compared with no or rare consumption [13,14]. Occupational exposure to wood dust is estimated to be associated with a 50% increased risk of NPC [15].

### Research Significance and Objective

Given the strong association of NPC with its modifiable risk factors and the changing epidemiological profile due to trends in NPC incidence and mortality, understanding the current epidemiological profile of international variations in NPC incidence and mortality is essential. This would allow public health policy makers to make evidence-based decisions for primary prevention and optimize the allocation of resources to reduce the global burden of NPC. Considering the growing and aging global population, predicting the future NPC burden is vital for better planning of future cancer control programs. We therefore examined the geographic variations in NPC incidence and mortality across world regions and countries worldwide based on the GLOBOCAN estimates for the year 2020 and predict the future NPC burden up to 2040 based on demographic projections.

## Methods

### Data Sources

The numbers of new cases of and deaths from NPC (International Classification of Diseases, tenth revision C11) were extracted from the GLOBOCAN 2020 database for 185 countries or territories by sex and by 5-year age groups (0-4, 5-9, …, 80-84, 85 years and older) [16-18]. Corresponding population data for 2020 were retrieved from the United Nations website [19]. The population projections used in this study are based on the future fertility rates [19]. The data sources and methods used in compiling the global cancer estimates for 2020 have been described in detail elsewhere [17]. Briefly, the GLOBOCAN national estimates are dependent on the availability of recorded high-quality national and subnational incidence (from population-based cancer registries) and national mortality data (from vital registration systems) [17]. Nine methods were employed depending on the best available data on cancer-specific incidence or mortality data [17]. The hierarchical set of methods includes utilization of short-term and long-term prediction models based on historical observed data, estimated incidence, mortality based on modelled survival, approximation using observed data at the subnational level, or data from neighboring countries [17]. In countries where national mortality data were available but national or subnational cancer registries were not, national incidence estimation relied on national mortality estimates and modelling of the mortality to incidence ratio from neighboring countries [17]. In countries where neither mortality nor incidence data were available, incidence and mortality estimates were based on mortality or incidence data of neighboring countries [17]. Thus, the validity of national cancer incidence and mortality estimates is dependent on the degree of representativeness and the quality of the source information [17]. The methods used to derive the 2020 estimates correspond to those used previously for 2018, 2012, and 2008 [20-22].

### Statistical Analysis

We present tables and figures of the estimated new cases and deaths as well as 2 summary measures by using direct standardization, namely, the age-standardized incidence rates (ASIRs) and age-standardized mortality rates (ASMRs) per 100,000 person-years based on the 1966 Segi–Doll World standard population [23,24] and the cumulative risk of being diagnosed with or dying from NPC before the age of 75 years, assuming the absence of competing causes of death [25]. We predicted the future number of NPC cases and deaths worldwide by the United Nation’s 4-tier Human Development Index (HDI), where HDI was used to assess the cancer burden at varying levels of development (low, medium, high, and very high HDI) [26] and in China up to the year 2040 based on demographic projections and scenarios of annually increasing (+1%, +2%, +3%, +4%), stable (0%), or decreasing (–1%, –2%, –3%, –4%) rates from the baseline year of 2020. We did not use scenarios of rates changing by SD 5% or more because such changes would be unlikely to occur in real life [4,5]. Predictions were calculated by applying the age-specific rates for the year 2020 (and each of the increasing or decreasing scenarios described) to the corresponding projected population data as estimated by the United Nations Development Program. The results are presented by country and aggregated across 20 United Nations–defined world regions [19] and according to the HDI group in 2020. Data management and analyses were performed in R software (version 4.0.2; R Foundation for Statistical Computing) [27]. Figures were plotted using SigmaPlot software (version 12.5; Systat Software Inc) [28]. Global maps of NPC incidence and mortality rates by country are shown using R software (version 4.0.2; R Foundation for Statistical Computing) [27].

### Ethical Considerations

This study does not involve human participants and animals. Ethics approval was not required for this study, as this study used existing nonidentifiable data that were aggregated at the population level.

## Results

### Global Burden of NPC Incidence and Mortality

In 2020, an estimated 133,354 people were diagnosed with NPC worldwide, corresponding to an ASIR of 1.5 per 100,000 person-years (Table 1). More men (96,371 cases) than women (36,983 cases) were diagnosed with NPC, and the ASIRs in men were approximately 3-fold higher than those in women (2.2 vs 0.8 per 100,000 person-years, respectively) (Multimedia Appendix 1). Globally, an estimated 80,008 people died from NPC, corresponding to an ASMR of 0.9 per 100,000 person-years (Table 1). Mortality in men was also higher than that in women, with 58,094 and 21,914 deaths, corresponding to ASMRs of 1.3 and 0.5 per 100,000 person-years, respectively (Multimedia Appendix 1). In addition, the cumulative risk of being diagnosed with and dying from NPC before the age of 75 years was 1 in 476 (0.21%) and 1 in 667 (0.15%), respectively (Table 1).

**Table 1:** Nasopharyngeal cancer incidence and mortality in both sexes combined in 2020 by world region and human development index level.

		Population (N=7,794,799)	Incidence (n=133,354)				Mortality (n=80,008)		
		Values (in thousands), n (%)	Cases, n (%)	ASIR^a^	Cumulative risk^b^	Deaths, n (%)		ASMR^c^	Cumulative risk^b^
**Europe**									
	Northern Europe	106,261 (1.4)	415 (0.3)	0.26	0.04	247 (0.3)		0.12	0.03
	Western Europe	196,146 (2.5)	1304 (1)	0.40	0.06	502 (0.6)		0.12	0.03
	Southern Europe	153,423 (2)	1584 (1.2)	0.64	0.09	746 (0.9)		0.24	0.05
	Central and Eastern Europe	293,013 (3.8)	1901 (1.4)	0.43	0.06	1091 (1.4)		0.22	0.04
**America**									
	Northern America	368,870 (4.7)	2177 (1.6)	0.41	0.06	1071 (1.3)		0.16	0.04
	South America	430,760 (5.5)	1423 (1.1)	0.28	0.05	797 (1)		0.15	0.03
	Central America	179,670 (2.3)	309 (0.2)	0.17	0.03	181 (0.2)		0.10	0.02
	Caribbean	43,532 (0.6)	313 (0.2)	0.56	0.10	198 (0.3)		0.34	0.07
**Asia**									
	Eastern Asia	1,678,090 (21.5)	65,866 (49.4)	2.70	0.35	36,453 (45.6)		1.40	0.23
	China	1,447,470 (18.6)	62,444 (46.8)	3.00	0.40	34,810 (43.5)		1.60	0.27
	South-Central Asia	2,014,709 (25.8)	8366 (6.3)	0.43	0.08	6117 (7.7)		0.32	0.06
	South-Eastern Asia	668,620 (8.6)	36,747 (27.6)	5.00	0.75	24,219 (30.3)		3.30	0.59
	Western Asia	278,429 (3.6)	2680 (2)	1.00	0.15	1645 (2.1)		0.63	0.11
**Oceania**									
	Australia and New Zealand	30,322 (0.4)	176 (0.1)	0.42	0.06	101 (0.1)		0.19	0.04
	Melanesia	11,123 (0.1)	22 (0.1)	0.25	0.03	14 (0.1)		0.17	0.02
	Micronesia/Polynesia	1233 (<0.1)	30 (0.1)	2.20	0.24	26 (0.1)		1.93	0.21
**Africa**									
	Northern Africa	246,233 (3.2)	3525 (2.6)	1.60	0.25	2113 (2.6)		0.98	0.20
	Western Africa	401,861 (5.2)	1906 (1.4)	0.70	0.10	1289 (1.6)		0.53	0.09
	Southern Africa	67,504 (0.9)	212 (0.2)	0.34	0.07	145 (0.2)		0.25	0.06
	Middle Africa	179,595 (2.3)	1212 (0.9)	1.10	0.15	852 (1.1)		0.86	0.13
	Eastern Africa	445,406 (5.7)	3186 (2.4)	1.10	0.20	2201 (2.8)		0.88	0.18
**Human Development Index**									
	Very high human development index	1,564,286 (20.1)	14,911 (11.2)	0.66	0.09	7828 (9.8)		0.30	0.05
	High human development index	2,909,468 (37.3)	93,153 (69.9)	2.50	0.35	54,850 (68.6)		1.40	0.24
	Medium human development index	2,327,556 (29.9)	19,543 (14.7)	0.89	0.15	13,314 (16.6)		0.62	0.12
	Low human development index	990,175 (12.7)	5722 (4.3)	0.89	0.15	3996 (5)		0.69	0.13
	World	7,794,799 (100)	133,354 (100)	1.50	0.21	80,008 (100)		0.88	0.15

^a^ASIR: age-standardized incidence rate per 100,000 person-years.

^b^Cumulative risk of being diagnosed with or dying from nasopharyngeal cancer before the age of 75 years in 2020.

^c^ASMR: age-standardized mortality rate per 100,000 person-years.

### Geographical Variations in NPC Incidence and Mortality by World Region

**Figure 1:**
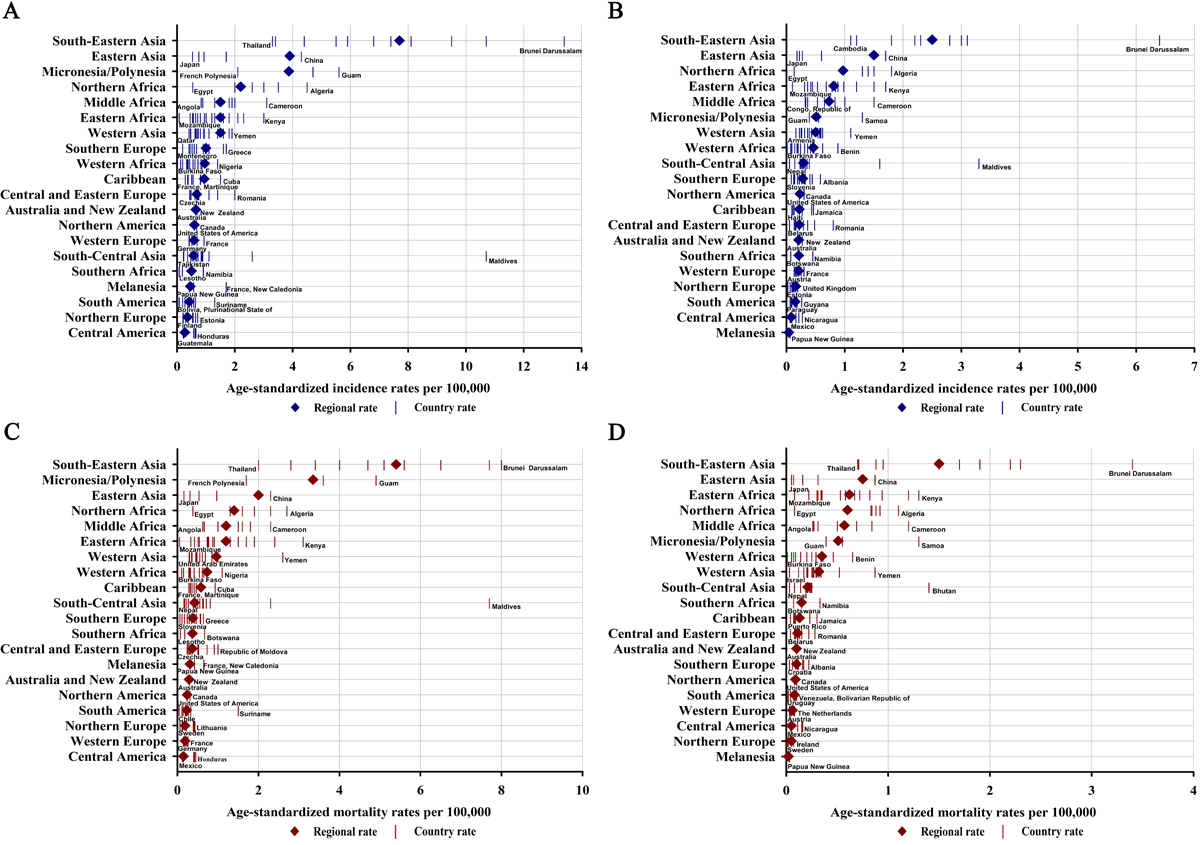
Age-standardized rates per 100,000 person-years of nasopharyngeal cancer incidence in (A) men and (B) women and mortality in (C) men and (D) women, which are ordered according to descending incidence rates by world region. Countries with zero cases are not shown in this figure.

The largest numbers of cases and deaths from NPC in both sexes combined were estimated in Eastern Asia (65,866/133,354, 49.39% of total NPC cases and 36,453/80,008, 45.56% of total NPC deaths), followed by South-Eastern Asia (36,747/133,354, 27.55% and 24,219/80,008, 30.15%, respectively) and South-Central Asia (8366/133,354, 6.27% and 6117/80,008, 7.64%, respectively) (Multimedia Appendix 2). A male predominance in NPC cases and deaths was consistent across all world regions (Multimedia Appendix 1). The incidence rates of NPC showed approximately 29-fold variation in men and 63-fold variation in women across world regions (Figure 1A, Figure 1B, and Multimedia Appendix 1). In men, the ASIR per 100,000 person-years was the highest in South-Eastern Asia (7.7), followed by Eastern Asia (3.9) and Micronesia/Polynesia (3.9), but the lowest ASIR was in Central America (0.3). In women, the highest ASIR per 100,000 person-years was detected in South-Eastern Asia (2.5), followed by Eastern Asia (1.5), and Northern Africa (1.0), and the lowest was found in Melanesia (less than 0.1). The mortality rates of NPC varied approximately by 36-fold in men and 75-fold in women among world regions (Figure 1C, Figure 1D, and Multimedia Appendix 1). In men, the highest ASMR per 100,000 person-years was detected in South-Eastern Asia (5.4), followed by Micronesia/Polynesia (3.4) and Eastern Asia (2.0), and the lowest was observed in Central America (0.2). In women, the highest ASMR per 100,000 person-years was found in South-Eastern Asia (1.5), followed by Eastern Asia (0.8) and Eastern Africa (0.6), and the lowest was seen in Melanesia (<0.1). Sex-related disparities were also noted, with NPC ASIRs and ASMRs higher in men than in women across world regions. For example, the male-to-female ASIR and ASMR ratios ranged from 1.9 and 1.9 in Eastern Africa to 11.5 and 15.5 in Melanesia, respectively.

### Geographical Variations in NPC Incidence and Mortality by Country

**Figure 2:**
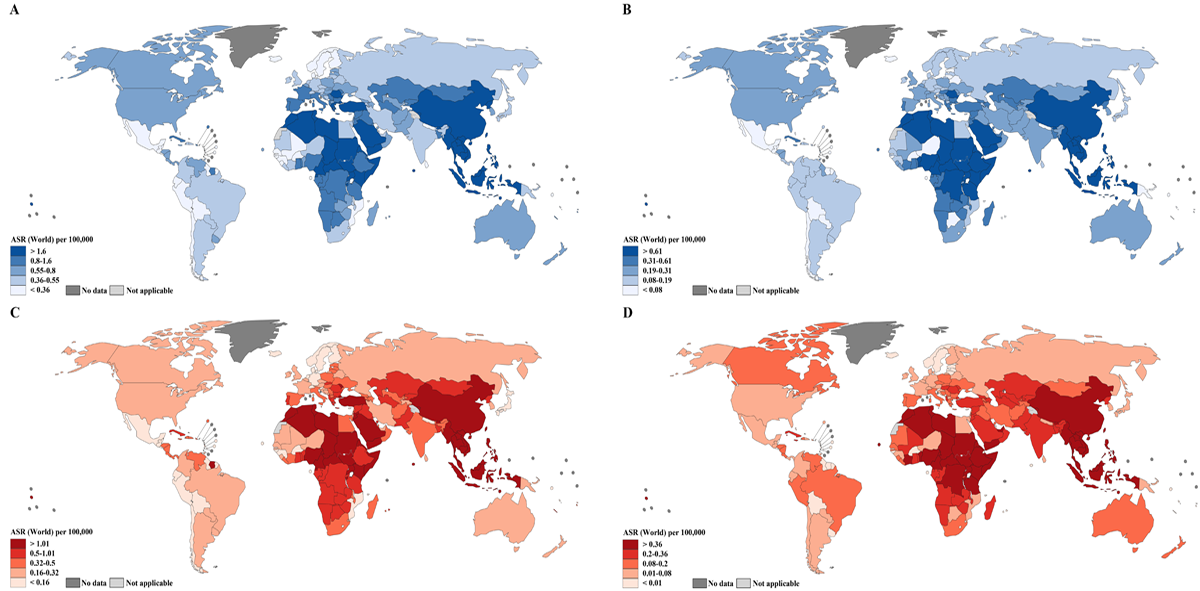
Global map of age-standardized rates of nasopharyngeal cancer incidence in (A) men and (B) women and mortality in (C) men and (D) women per 100,000 person-years by country. ASR: age-standardized rate.

At the national level, China (62,444/133,354, 46.82% of global NPC cases; 34,810/80,008, 43.50% of global NPC deaths) was the greatest contributor to the global NPC burden due to its large population (1,447,470,000/7,794,799,000, 18.56% of the global population) and high incidence and mortality rates (ASIR, 3.0 per 100,000 person-years; ASMR, 1.6 per 100,000 person-years) (Table 1). In terms of incidence rates, the highest ASIR per 100,000 person-years occurred in Brunei Darussalam (13.4), followed by Maldives (10.7) and Indonesia (10.7), in men and in Brunei Darussalam (6.4), followed by Maldives (3.3) and Malaysia (3.1), in women (Figure 1A, Figure 1B, Figure 2A, and Figure 2B). Notably, the incidence rates varied markedly within world regions. For example, in the high-risk region South-Eastern Asia, the ASIRs ranged from 3.3 per 100,000 person-years in Thailand to 13.4 per 100,000 person-years in Brunei Darussalam (high-to-low ASIR ratio = 4.1) in men and from 1.1 in Cambodia to 6.4 in Brunei Darussalam (high-to-low ASIR ratio = 5.8) in women (Figure 1A and Figure 1B). In terms of mortality rates, the highest ASMR per 100,000 person-years in men was found in Brunei Darussalam (8.0), followed by Maldives (7.7) and Indonesia (7.7), and the highest ASMR per 100,000 person-years in women was found in Brunei Darussalam (3.4), followed by Timor-Leste (2.3) and Lao People's Democratic Republic (2.2) (Figure 1C, Figure 1D, Figure 2C, and Figure 2D). Considerable variations in NPC mortality were also evident within world regions. For example, in South-Eastern Asia, the ASMRs per 100,000 person-years ranged from 2.0 in Thailand to 8.0 in Brunei Darussalam (high-to-low ASMR ratio = 4.0) in men and from 0.7 in Thailand to 3.4 in Brunei Darussalam (high-to-low ASMR ratio = 4.9) in women (Figure 1C and Figure 1D).

### NPC Incidence and Mortality by Level of Human Development

By HDI group, the vast majority of NPC cases and deaths occurred among 37.32% (2,909,468,000/7,794,799,000) of the world population living in high HDI countries, representing 69.85% (93,153/133,354) of new cases and 68.55% (54,850/80,008) of deaths globally (Table 1). Both ASIRs and ASMRs were the highest in high HDI countries, with the rates being 2.5 and 1.4 per 100,000 person-years, respectively. Notably, both the lowest ASIRs and ASMRs were observed in very high HDI countries. The ASIRs and ASMRs in low HDI countries were similar to those in medium HDI countries.

### Predicted Number and Percentage Increase of Cases and Deaths From NPC

**Figure 3:**
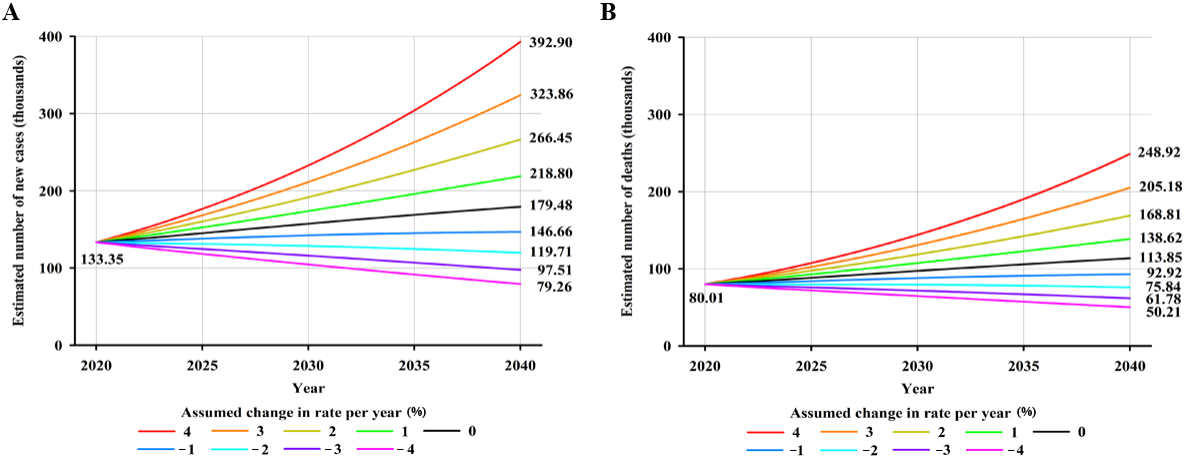
Predicted number of (A) new cases and (B) deaths from nasopharyngeal cancer, assuming 9 scenarios of annual change in global rates between 2020 and 2040, both sexes combined. Nine scenarios include annually increasing (+1%, +2%, +3%, +4%), stable (0%), and decreasing (–1%, –2%, –3%, –4%) rates from the baseline year of 2020.

**Figure 4:**
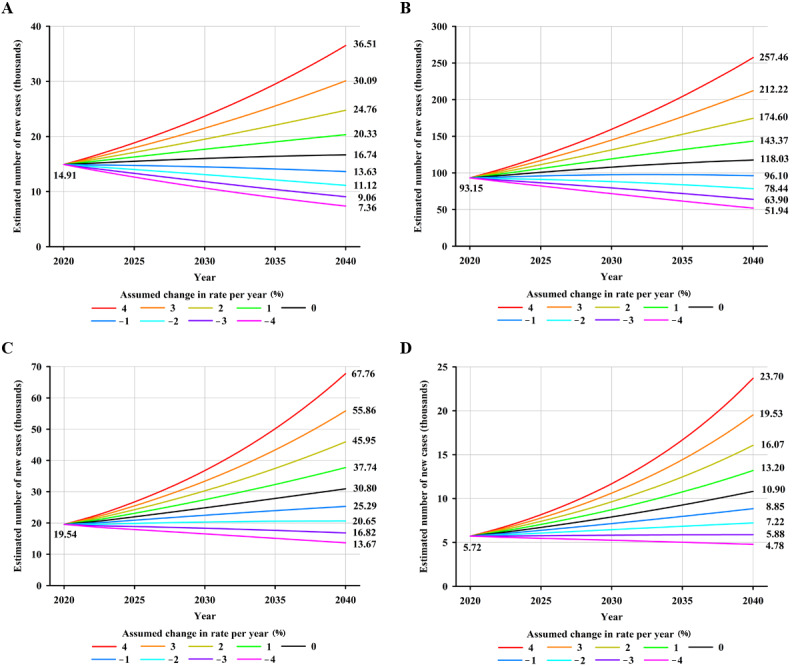
Predicted number of new nasopharyngeal cancer cases, assuming 9 scenarios of annual change in rates between 2020 and 2040, in (A) countries with very high human development index, (B) countries with high human development index, (C) countries with medium human development index, and (D) countries with low human development index, both sexes combined. Nine scenarios consist of annually increasing (+1%, +2%, +3%, +4%), stable (0%), and decreasing (–1%, –2%, –3%, –4%) rates from the baseline year of 2020.

**Figure 5:**
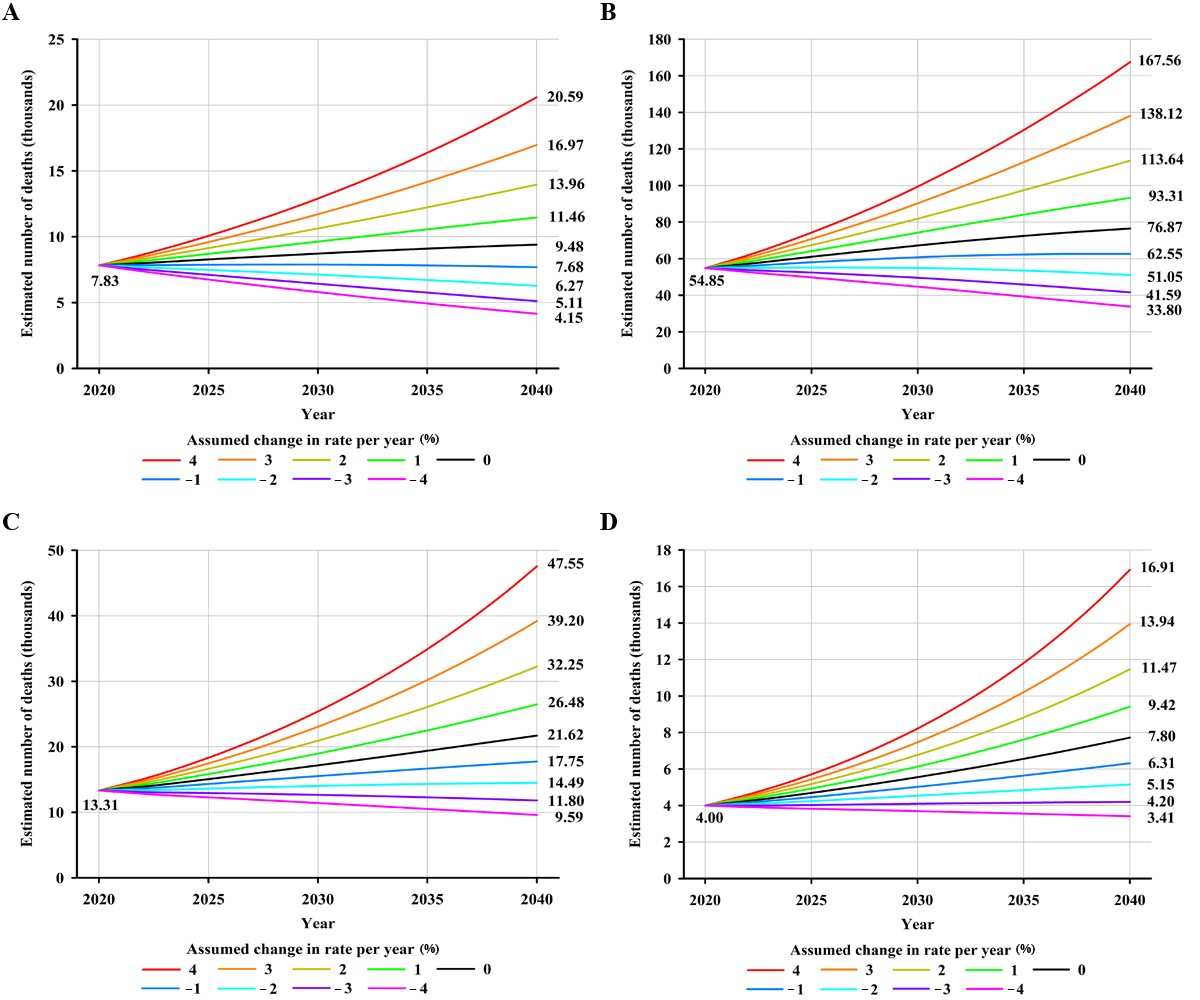
Predicted number of deaths from nasopharyngeal cancer assuming 9 scenarios of annual change in rates between 2020 and 2040 in (A) countries with very high human development index, (B) countries with high human development index, (C) countries with medium human development index, and (D) countries with low human development index, both sexes combined. Nine scenarios consist of annually increasing (+1%, +2%, +3%, +4%), stable (0%), and decreasing (–1%, –2%, –3%, –4%) rates from the baseline year of 2020.

Worldwide, an estimated 179,476 new NPC cases are projected to occur in 2040—an increase of approximately 34.58% (46,122/133,354)—compared with 133,354 cases in 2020, assuming that global incidence rates in 2020 remain stable (Figure 3A). Moreover, a 2% annual increase in incidence rates from 2020 would more than double the total annual NPC cases by 2040 (Figure 3A). Notably, it would require a 2% annual decrease in mortality rates to ensure that there would be fewer NPC cases in 2040 (119,710 cases) than there were in 2020 (133,354 cases). In terms of mortality, NPC deaths were estimated to increase by approximately 42.29% (33,843/80,008), from 80,008 in 2020 to 113,851 in 2040, assuming that mortality rates in 2020 remained unchanged (Figure 3B). An annual decline of 2% in mortality rates would be required to achieve fewer NPC deaths in 2040 compared to the estimated deaths in 2020 (Figure 3B). By HDI group, the largest absolute increase in NPC cases and deaths is estimated to occur in high HDI countries, with 26.7% (24,874/93,153) more cases (24,874 additional cases) and 40.15% (22,024/54,850) more deaths (22,024 additional deaths) per year by 2040 (Multimedia Appendix 3), assuming that incidence and mortality rates in 2020 remain constant, reflecting the already high rates in high HDI countries and its large population, which will continue to grow. However, the greatest relative increases in cases and deaths will occur in low HDI countries (5181/5722, 90.54% and 3808/3996, 95.29% increase, respectively) (Multimedia Appendix 3). Notably, decreases in both incidence and mortality rates would need to be greater than 4%, 3%, 2%, and 1% to retain the predicted NPC burden in 2040 at the level observed in 2020 for low, medium, high, and very high HDI countries, respectively (Figures 4-5).

**Figure 6:**
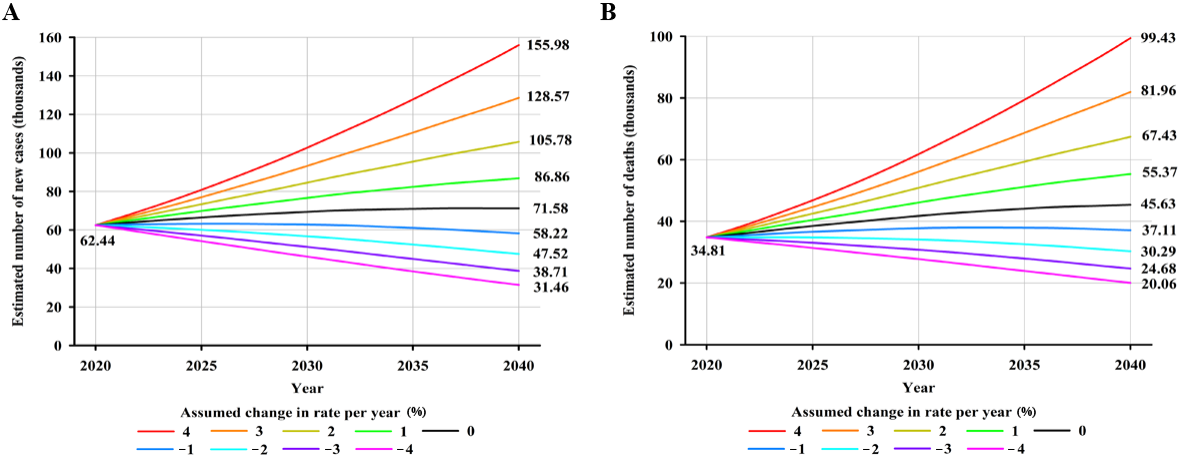
Predicted number of (A) new cases and (B) deaths from nasopharyngeal cancer assuming 9 scenarios of annual change in rates between 2020 and 2040 in China (both sexes combined). Nine scenarios consist of annually increasing (+1%, +2%, +3%, +4%), stable (0%), and decreasing (–1%, –2%, –3%, –4%) rates from the baseline year of 2020.

In China, the number of NPC cases was estimated to increase by approximately 13.99% (8742/62,444), from 62,444 in 2020 to 71,186 in 2040, assuming that incidence rates in 2020 remained stable (Figure 6A). Similarly, NPC deaths are set to increase by close to 30.35% (10,568/34,810) based on demographic changes alone, from 34,810 in 2020 to 45,378 in 2040 (Figure 6B). As an illustration, it would take greater than 1% and 2% decline in the annual incidence and mortality rate from 2020 to 2040 to achieve fewer cases and deaths in 2040 compared to the level seen in 2020, respectively.

## Discussion

### Principal Findings

Globally, approximately 133,354 new NPC cases were reported and 80,008 deaths occurred in 2020 [16-19]. The largest numbers of cases and deaths of NPC were estimated in Eastern Asia, in which China contributed most to this burden [29]. NPC incidence and mortality rates in men were substantially higher than those in women across all world regions. The highest incidence rates for both men and women were detected in South-Eastern Asia and Eastern Asia, and the mortality rates for both men and women were the highest in South-Eastern Asia. The number of cases and deaths from NPC will increase by more than 35% over the next 20 years worldwide due to population growth and aging alone, with annual NPC cases and deaths expected to almost double in low HDI countries by 2040 [26].

### Interpretation of the Disparities of NPC Burden by Geography and by Gender

The changing epidemiological profile and the disparities of NPC burden by geography and by gender might be largely associated with differences in the prevalence of its risk factors and improvement of diagnostic and treatment techniques. Previous studies have shown that NPC incidence and mortality rates have significantly decreased in most European, Northern American, and Eastern and South-Eastern Asian countries over the past decades [4,5]. The declining trends in NPC incidence rates in Europe and Northern America might be related to decreased tobacco smoking prevalence, and the declines in NPC incidence rates in Eastern and South-Eastern Asia might be explained by the decreased intake of salted fish and preserved food [4], while decreases in mortality rates might also be the result of advances in diagnostic (eg, diagnostic imaging accuracy) and radiotherapy techniques (eg, the introduction of intensity-modulated radiation therapy) [5]. In particular, the reduction of NPC burden in China is partly due to the implementation of EBV screening for early detection of NPC since the 1970s [29].

The relatively higher NPC burden in Eastern and South-Eastern Asian countries could be largely related to the high prevalence of EBV infection [30]. In addition, 316 million adults are estimated to smoke in China, who account for nearly one-third of the Chinese who smoke and contribute to 40% of the tobacco consumption worldwide [31]. The higher NPC burden in China could therefore be partly explained by its large tobacco consumption and production. The relatively larger consumption of salt-preserved foods in most Eastern and South-Eastern Asian and Northern African countries might also explain part of the remarkable international variations of NPC burden [2]. The elevated NPC burden observed in men compared with that observed in women across all regions can be partly explained by the higher smoking prevalence and occupational exposure to carcinogens [32,33]. In addition, the decreased NPC burden in women could be related to the potentially protective effect of endogenous estrogens [33,34]. Of note, genetics might also play an important role in the pathogenesis of NPC. Previous studies showed that NPC incidence in Southern China is about 20-50 times higher than that in western countries [1-3]. Despite immigration of second- and third-generation Chinese people to western countries, they are still at a higher risk for NPC, thereby demonstrating a sustained risk for NPC even after changing environments [35,36].

### Interpretation of the Projected Number of Cases and Deaths From NPC

Our findings show that the projected number of cases and deaths from NPC are expected to increase to 179,476 and 113,851 by 2040 worldwide, respectively, as a result of population growth and aging alone. A 2% annual decrease in global incidence and mortality rates would be needed to halt the increasing NPC burden by 2040. Notably, our findings show that even larger decreases in incidence and mortality rates would be required to reduce the future NPC burden in low and medium HDI countries. Considering these changes, the reallocation of resources for NPC primary prevention programs aimed at reducing population levels of EBV infection, tobacco smoking, intake of salted fish and other salt-preserved food, and occupational exposure to NPC carcinogens in certain regions and countries and the increased access to early detection modalities (eg, NPC screening) and health care services for high-risk populations identified by our study are crucial for reducing the global NPC burden.

### Comparison With Prior Work

To the best of our knowledge, this study is the first to provide a comprehensive picture of the most up-to-date epidemiological profile of NPC incidence and mortality on a global scale based on the GLOBOCAN 2020 data set, which is highly relevant for cancer control and clinical practice. The numbers and rates of NPC presented in this study are estimates based on the best available data (reviewed for their completeness, coverage, and accuracy) from population-based cancer registries.

### Limitations

There are several limitations in this study. First, although our findings are based on the best available and high-quality data, caution is warranted when interpreting the findings for countries with limited coverage from population-based cancer registries and where proxy data were used to obtain national estimates [17]. Second, the GLOBOCAN estimates did not account for the impact of the COVID-19 pandemic on cancer diagnoses because the GLOBOCAN estimates were based on extrapolations of previous years of cancer data [17]. Third, the projections of the future burden of NPC in 2040 considered neither the recent changes in NPC incidence and mortality rates nor the heterogeneity in NPC incidence and mortality trends among countries. Thus, the predictions in our study likely represent an overestimate of the future NPC burden, given the declining trends of NPC incidence and mortality rates in recent decades worldwide [4,5] and should be interpreted with caution. Finally, we could not distinguish the required changes in the modifiable risk factors and their corresponding effects on predicted NPC incidence and mortality rates, which suggests the need for further studies to clarify the impact of changes in exposure to modifiable risk factors on the burden of NPC.

### Conclusions

NPC remains a considerable public health challenge worldwide. NPC incidence and mortality vary markedly across world regions and countries and between men and women, and this variation is likely related to differences in EBV infection, tobacco smoking, intake of salted fish and other salt-preserved food, and occupational exposure to NPC carcinogens. Given the remarkable geographic disparity of NPC burden across world regions and countries, this study highlights the urgent need to formulate more effective primary NPC prevention strategies and prioritize allocation of NPC prevention and treatment resources for high-risk populations to tackle the NPC burden in certain regions and countries.

## Appendix

Multimedia Appendix 1 Nasopharyngeal cancer incidence and mortality in 2020 by sex, world region, and human development index level.

Multimedia Appendix 2 Distribution of (A) nasopharyngeal cancer cases and (B) deaths by world region in 2020, both sexes combined.

Multimedia Appendix 3 Predicted percentage change (absolute numbers are shown above bars) of (A) new cases and (B) deaths from nasopharyngeal cancer (both sexes combined) between 2020 and 2040 globally and by HDI, assuming that incidence rates and mortality rates in 2020 remained unchanged. HDI: Human Development Index.

## Abbreviations

ASIR: age-standardized incidence rate
ASMR: age-standardized mortality rate
EBV: Epstein-Barr virus
HDI: Human Development Index
NPC: nasopharyngeal cancer

## Data Availability

Data are available in the Global Cancer Observatory, a public open access repository [37]. Processed data sets used in this analysis are available from the corresponding author upon reasonable request.
