# Supplementary material for: Nasopharyngeal Cancer Incidence and Mortality in 185 Countries in 2020 and the Projected Burden in 2040: Population-Based Global Epidemiological Profiling
Source: JMIR Public Health Surveill. 2023 Sep 20;9:e49968. doi: 10.2196/49968 (PMC10551785; doi:10.2196/49968)
Supplement: Multimedia Appendix 1 [file publichealth_v9i1e49968_app1.docx]

**Multimedia Appendix 1.** Nasopharyngeal cancer incidence and mortality in 2020 by sex, world region, and human development index level.

|  | | Incidence | | | | | | | Mortality | | | | | | |
| --- | --- | --- | --- | --- | --- | --- | --- | --- | --- | --- | --- | --- | --- | --- | --- |
|  | | Males | | | Females | | | M:F^a^ rate ratio | Males | | | Females | | | M:F rate ratio |
|  | | Cases, n (%) | ASIR^b^ | Cum. risk^c^ | Cases, n (%) | ASIR^b^ | Cum. risk^c^ |  | Deaths, n (%) | ASMR^d^ | Cum. risk^c^ | Deaths, n (%) | ASMR^d^ | Cum. risk^c^ |  |
| **Europe** | | | | | | | | | | | | | | | |
|  | Northern Europe | 287 (0.3) | 0.36 | 0.05 | 128 (0.4) | 0.16 | 0.02 | 2.3 | 187 (0.3) | 0.19 | 0.05 | 60 (0.3) | 0.05 | 0.01 | 3.8 |
|  | Western Europe | 936 (1.0) | 0.59 | 0.09 | 368 (1.0) | 0.21 | 0.03 | 2.8 | 366 (0.6) | 0.19 | 0.04 | 136 (0.6) | 0.06 | 0.01 | 3.2 |
|  | Southern Europe | 1230 (1.3) | 1.00 | 0.14 | 354 (1.0) | 0.28 | 0.04 | 3.6 | 561 (1.0) | 0.38 | 0.08 | 185 (0.8) | 0.10 | 0.02 | 3.8 |
|  | Central and Eastern Europe | 1342 (1.4) | 0.68 | 0.10 | 559 (1.5) | 0.22 | 0.03 | 3.1 | 783 (1.4) | 0.37 | 0.07 | 308 (1.4) | 0.11 | 0.02 | 3.4 |
| **America** | | | | | | | | | | | | | | | |
|  | Northern America | 1555 (1.6) | 0.60 | 0.09 | 622 (1.7) | 0.23 | 0.03 | 2.6 | 757 (1.3) | 0.24 | 0.05 | 314 (1.4) | 0.09 | 0.02 | 2.7 |
|  | South America | 993 (1.0) | 0.42 | 0.08 | 430 (1.2) | 0.15 | 0.03 | 2.8 | 552 (1.0) | 0.23 | 0.05 | 245 (1.1) | 0.08 | 0.02 | 2.9 |
|  | Central America | 232 (0.2) | 0.27 | 0.06 | 77 (0.2) | 0.08 | 0.01 | 3.4 | 132 (0.2) | 0.15 | 0.03 | 49 (0.2) | 0.05 | 0.01 | 3.0 |
|  | Caribbean | 247 (0.3) | 0.94 | 0.18 | 66 (0.2) | 0.22 | 0.04 | 4.3 | 157 (0.3) | 0.58 | 0.13 | 41 (0.2) | 0.13 | 0.03 | 4.5 |
| **Asia** | | | | | | | | | | | | | | | |
|  | Eastern Asia | 47,857 (49.7) | 3.90 | 0.52 | 18,009 (48.7) | 1.50 | 0.19 | 2.6 | 26,345 (45.4) | 2.00 | 0.34 | 10108 (46.1) | 0.75 | 0.13 | 2.7 |
|  | China | 45,331  (47.0) | 4.30 | 0.59 | 17,113  (46.3) | 1.70 | 0.23 | 2.5 | 25,118  (43.2) | 2.30 | 0.39 | 9692  (44.2) | 0.87 | 0.15 | 2.6 |
|  | South-Central Asia | 5611 (5.8) | 0.57 | 0.10 | 2755 (7.5) | 0.29 | 0.06 | 2.0 | 4111 (7.1) | 0.42 | 0.07 | 2006 (9.2) | 0.21 | 0.05 | 2.0 |
|  | South-Eastern Asia | 27,361 (28.4) | 7.70 | 1.20 | 9386 (25.4) | 2.50 | 0.37 | 3.1 | 18,521 (31.9) | 5.40 | 0.97 | 5698 (26.0) | 1.50 | 0.28 | 3.6 |
|  | Western Asia | 2013 (2.1) | 1.50 | 0.24 | 667 (1.8) | 0.50 | 0.07 | 3.0 | 1236 (2.1) | 0.96 | 0.18 | 409 (1.9) | 0.32 | 0.05 | 3.0 |
| **Oceania** | | | | | | | | | | | | | | | |
|  | Australia and New Zealand | 134 (0.1) | 0.65 | 0.09 | 42 (0.1) | 0.21 | 0.03 | 3.1 | 73 (0.1) | 0.29 | 0.06 | 28 (0.1) | 0.10 | 0.02 | 2.9 |
|  | Melanesia | 20 (<0.1) | 0.46 | 0.05 | 2 (<0.1) | 0.04 | <0.01 | 11.5 | 13 (<0.1) | 0.31 | 0.04 | 1 (<0.1) | 0.02 | 0.00 | 15.5 |
|  | Micronesia/Polynesia | 26 (<0.1) | 3.87 | 0.45 | 4 (<0.1) | 0.51 | 0.03 | 4.9 | 22 (<0.1) | 3.35 | 0.39 | 4 (<0.1) | 0.51 | 0.03 | 4.0 |
| **Africa** | | | | | | | | | | | | | | | |
|  | Northern Africa | 2399 (2.5) | 2.20 | 0.39 | 1126 (3.0) | 0.97 | 0.14 | 2.3 | 1444 (2.5) | 1.40 | 0.30 | 669 (3.1) | 0.60 | 0.11 | 2.3 |
|  | Western Africa | 1253 (1.3) | 0.96 | 0.13 | 653 (1.8) | 0.46 | 0.07 | 2.1 | 845 (1.5) | 0.73 | 0.11 | 444 (2.0) | 0.35 | 0.07 | 2.1 |
|  | Southern Africa | 140 (0.2) | 0.50 | 0.10 | 72 (0.2) | 0.21 | 0.05 | 2.4 | 95 (0.2) | 0.37 | 0.09 | 50 (0.2) | 0.15 | 0.04 | 2.5 |
|  | Middle Africa | 765 (0.8) | 1.50 | 0.22 | 447 (1.2) | 0.73 | 0.09 | 2.1 | 536 (0.9) | 1.20 | 0.19 | 316 (1.4) | 0.57 | 0.07 | 2.1 |
|  | Eastern Africa | 1970 (2.0) | 1.50 | 0.29 | 1216 (3.3) | 0.81 | 0.13 | 1.9 | 1358 (2.3) | 1.20 | 0.26 | 843 (3.9) | 0.62 | 0.12 | 1.9 |
| **Human Development Index** | | | | | | | | | | | | | | | |
|  | Very high HDI^e^ | 11,052 (11.5) | 1.00 | 0.14 | 3859 (10.4) | 0.34 | 0.05 | 2.9 | 5874 (10.1) | 0.48 | 0.09 | 1954 (8.9) | 0.14 | 0.03 | 3.4 |
|  | High HDI | 68,301 (70.9) | 3.70 | 0.53 | 24,852 (67.2) | 1.30 | 0.19 | 2.8 | 40,534 (69.8) | 2.20 | 0.38 | 14,316 (65.3) | 0.73 | 0.13 | 3.0 |
|  | Medium HDI | 13,407 (13.9) | 1.20 | 0.20 | 6136 (16.6) | 0.56 | 0.11 | 2.1 | 9159 (15.8) | 0.86 | 0.15 | 4155 (19.0) | 0.38 | 0.08 | 2.3 |
|  | Low HDI | 3587 (3.7) | 1.20 | 0.20 | 2135 (5.8) | 0.62 | 0.10 | 1.9 | 2508 (4.3) | 0.93 | 0.18 | 1488 (6.8) | 0.47 | 0.09 | 2.0 |
|  | World | 96,371 (100.0) | 2.20 | 0.31 | 36,983 (100.0) | 0.82 | 0.12 | 2.7 | 58,094 (100.0) | 1.30 | 0.22 | 21,914 (100.0) | 0.47 | 0.08 | 2.8 |

^a^M:F: male:female.

^b^ASIR: age-standardized incidence rate per 100,000 person-years.

^c^Cumulative risk of being diagnosed with or dying from nasopharyngeal cancer until 74 years of age in 2020.

^d^ASMR: age-standardized mortality rate per 100,000 person-years.

^e^HDI: Human Development Index.
