# Supplementary figures and images for: Nasopharyngeal Cancer Incidence and Mortality in 185 Countries in 2020 and the Projected Burden in 2040: Population-Based Global Epidemiological Profiling
Source: JMIR Public Health Surveill. 2023 Sep 20;9:e49968. doi: 10.2196/49968 (PMC10551785; doi:10.2196/49968)

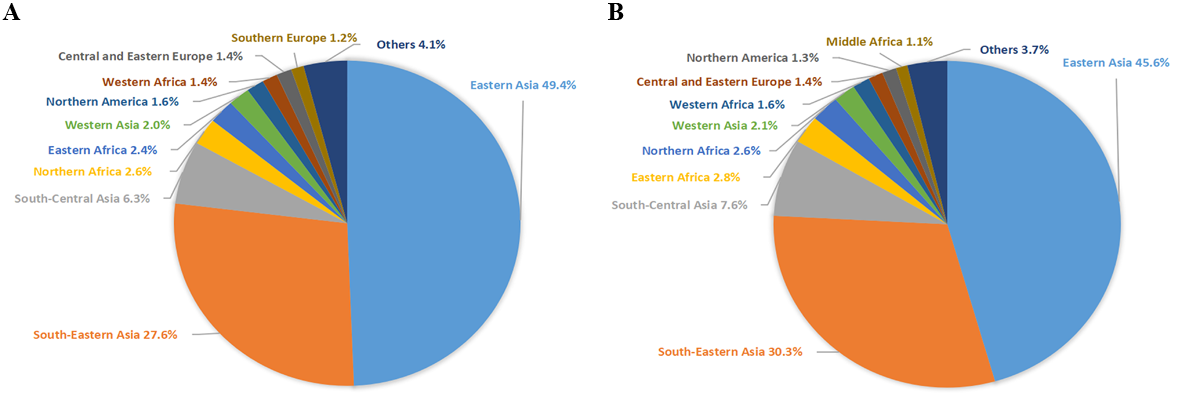

Supplement: Multimedia Appendix 2 [file publichealth_v9i1e49968_app2.png]

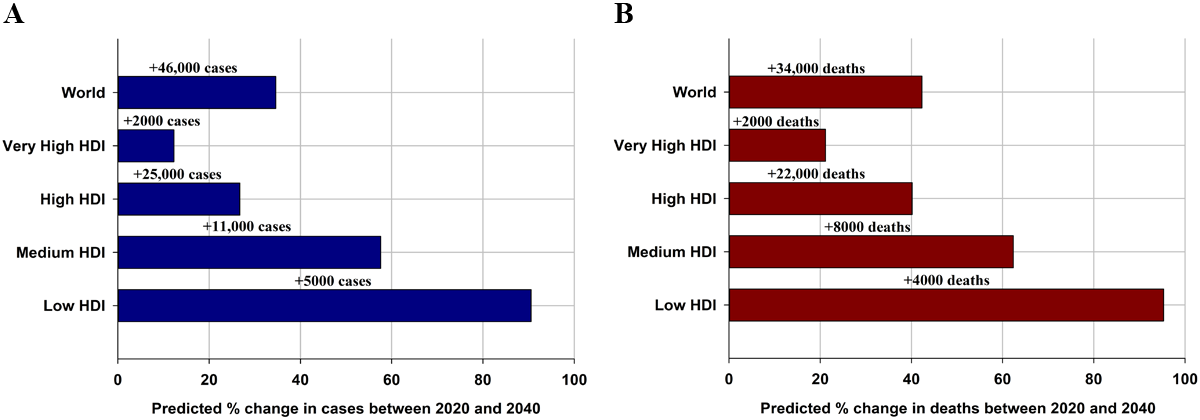

Supplement: Multimedia Appendix 3 [file publichealth_v9i1e49968_app3.png]
